# Limitations of Gene Duplication Models: Evolution of Modules in Protein Interaction Networks

**DOI:** 10.1371/journal.pone.0035531

**Published:** 2012-04-18

**Authors:** Frank Emmert-Streib

**Affiliations:** Computational Biology and Machine Learning Lab, Center for Cancer Research and Cell Biology, School of Medicine, Dentistry and Biomedical Sciences, Queen's University Belfast, Belfast, United Kingdom; University of Alberta, Canada

## Abstract

It has been generally acknowledged that the module structure of protein interaction networks plays a crucial role with respect to the functional understanding of these networks. In this paper, we study evolutionary aspects of the module structure of protein interaction networks, which forms a mesoscopic level of description with respect to the architectural principles of networks. The purpose of this paper is to investigate limitations of well known *gene duplication models* by showing that these models are lacking crucial structural features present in protein interaction networks on a mesoscopic scale. This observation reveals our incomplete understanding of the structural evolution of protein networks on the module level.

## Introduction

The understanding of evolutionary processes is not only of great interest to reconstruct the history of organic life and its evolution but can also help to shed light on the molecular functioning of organisms [1]–[6]. With the availability of large-scale sequence information and protein structures, the information stored in these entities could be systematically exploited with the help of computational and statistical methods [7]–[12]. Such studies have in common that a functional understanding is usually not obtained by direct investigations of molecular interactions but by inductive reasoning based on a comparative analysis. This is in contrast to studies based on the analysis of gene networks [13], because with the advent of network biology [14] and the availability of genome-scale networks, evolutionary questions can be addresses on the network-level [15]. Due to the fact that the structure of gene networks, e.g., metabolic, protein, or transcriptional regulatory networks, represent causal molecular interactions, direct studies of the biological function are enabled [16]–[18].

Since the introduction of random networks in the 1950s [19], [20] many new network classes have been invented [21]–[24], commonly called complex networks, and shown to provide better models for numerous natural phenomena [25]–[27]. Over the years, the interest in these complex networks has been gradually shifted from studying local properties, e.g., degree distributions, toward larger substructures or subnetworks forming motifs or communities [28]–[31]. In biology, the rational for this shift lies in the opportunity that gene networks offer in revealing insights about functional working mechanisms of a cell, if studied appropriately [13], [32], [33]. Similarly, this trend can be also observed in studies of the structural evolution of gene networks [34], [35].

The major purpose of this paper is to study two biologically motivated models that have been introduced to describe the evolution of protein-protein interaction (PPI) networks. More precisely, we study the question if the *network gene duplication* (NGD) model [36], [37] and the *duplication-mutation complementation* (DMC) model [38] resemble the module structure of biological protein interaction networks. Due to the similarity of both models, as described in detail in section ‘Network data and models’, we use the term *gene duplication model* (GDM) to either indicate the *network gene duplication* model or the *duplication-mutation complementation* model. The general idea of our study is to probe these evolutionary models by comparing networks generated with these models with biological PPI networks from various organisms [39], [40] to gain a deeper understanding of their capabilities. Here, the fact that a *gene duplication model* may have limitations would be of no surprise, since every model is merely an abstraction of reality sacrificing certain aspects to gain a mathematical representation. However, it is important to understand what *specific* limitations a *gene duplication model* is suffering from to judge its usefulness to serve as a model for the evolution of protein interaction networks. Considering the results from investigations studying either the NGD model or the DMC model with respect to the degree distribution of the networks, it appears unlikely that on this level of description refuting information can be found. Instead, in this article we are focusing on mesoscopic properties of networks in form of *modules* [41], [42]. The motivating idea for choosing this level is not only the fact that the module structure of networks is by far less well studied compared to the degree distribution, but, from a biological point of view, a module appears to be a more important entity with respect to the biological function of an organism than the degree of a gene. More specifically, genes and gene products establish by interacting with each other a biological function. This entity is of central importance to understand the functioning of an organism. In more abstract terms the interactions among genes establish an information flow that gives rise to this biological function. In this respect, modules can be considered to represent basic entities of information processing on a molecular level. Due to the fact that both, the *network gene duplication* model and the *duplication-mutation complementation* model, have been introduced to resemble gene inheritance [43], but not information processing in modules, the answer to the question if these models resemble the module structure of biological protein interaction networks does not immediately follow from their definition, but needs to be investigated. For reasons of completeness, we would like to mention that the two evolutionary models [36]–[38] are not the only models that has been proposed for the evolution of protein networks but there are a few other models, e.g., [44]–[46]. However, the NGD model and the DMC model investigated in this paper might be the most widely used and studied models in the literature.

In order to study the proposed question quantitatively, we pursue the following approach. First, we select an algorithm to identify the modules in networks. Second, we define several network-based measures that capture important information about the module structure of a network. These measures will form the components of a feature vector that represents the network. Third, we use agglomerative clustering to cluster the feature vectors in order to reveal similarities respectively differences between the clustered networks.

This paper is organized as follows. In the ‘Methods’ section we specify the network data we are analyzing and the methods we are applying. In the ‘Results’ section we present numerical results of our analysis and discuss our findings. The paper finishes with the ‘Discussion’ section presenting a summary and an outlook to future problems.

## Methods

In order to study the question if *gene duplication models* resemble the module structure of biological protein interaction networks, we need to realize that any *gene duplication model* (GDM) is formally a stochastic process [36]–[38]. That means if we generate two networks using the same model parameters, these networks will most likely not be identical. However, they share certain characteristics quantifiable by network-based measures, e.g., the exponent of their degree distribution or their edge density. This implies that a GDM, as any other stochastic process that generates networks, constitutes a network population or a network class. Throughout this paper, we use both terms synonymously. In the following we describe our general approach to study the population properties of a GDM.

**Figure 1:**
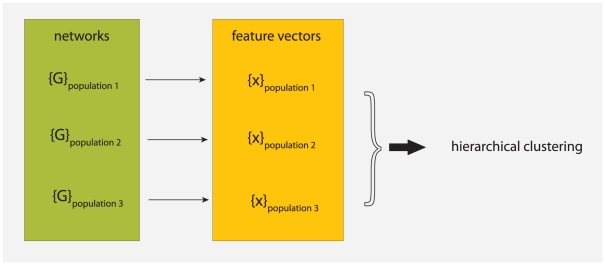
A schematic visualization of our approach to study properties of network populations. First, we map networks to feature vectors. Then we analysis these feature vectors with a hierarchical clustering. The resulting clustering allow us to conclude back to the similarity of the network populations.

Our overall approach is schematically visualized in Fig. 1. The basic idea is to map networks, which are part of a population, to feature vectors. That means the feature vectors are used as a representation of the networks, respectively the population of networks. We assume that there exists an underlying stochastic mechanism, or a model, that generates networks with common characteristics. These characteristics may vary from network to network because the underlying mechanism is stochastic rather than deterministic. The commonality of all networks generated from such a model forms a population. A specific example of a biologically motivated mechanism that generates protein networks is either the NGD model [36], [37] or the DMC model [38]. For a given set of model parameters these models establish a population of networks sharing common properties. Another example for such a mechanism is the preferential attachment model which generates scale-free networks [21]. In the following, we assume that also (biological) protein networks constitute a population which have been generated by evolutionary forces.

The quantitative analysis we will perform is based on the feature vectors derived from the networks. We conduct a comparative analysis applying a hierarchical clustering to investigate similarities between feature vectors. This allows us to conclude about the similarities of the underlying networks and, hence, about the similarities of the populations. We want to re-emphasize that our focus is on the properties of the network populations rather than on individual networks. This difference is crucial because we do not aim to derive results about individual networks but for the population.

### Finding modules

In recent years, many algorithms have been introduced for finding the community or module structure in networks which are based on a variety of different principles and approaches [47]–[50]. In this paper, we use the *edge-betweenness algorithm* introduced in [51] for finding the modules in the networks we study. This method is probably the best studied module algorithm. Together with the measure *modularity*, 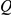, it has been widely used for analyzing biological networks [52]–[56]. The principle working mechanism of the *edge-betweenness algorithm* is to start from a connected network and remove successively edges with the highest *edge-betweenness* values, i.e., edges that occur on many shortest paths. The idea is that edges connecting separate modules are more likely to have high edge betweenness values because paths between modules must pass through them. Successively removing edges with the highest edge betweenness values results in a hierarchical tree of network components. The optimal partitioning of the network is obtained by finding the optimal cut of this tree. This is accomplished by using an optimization function, called *modularity* 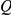. Application of this algorithm results in a non-overlapping module structure meaning that each node in the network is allowed to belong to exactly one module.

### Module measures

In order to characterize the modular structure of a network 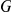, found by the application of a partitioning method 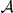, we use 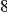 different measures introduced in the following. Some of these measures bear a resemblance to *indices* frequently used for the analysis of biological or chemical networks [57]–[61]. The motivation for the selection of the following measures is to obtain a heterogeneous set of network-based measures because we will use them as components of feature vectors.

**Figure 2:**
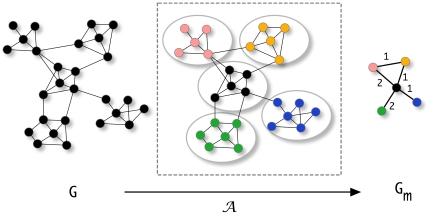
Mapping from the unweighted network 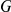 to the weighted network 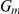 by application of a partitioning method 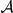. The numbers next to the edges of the module network 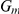 refer to the values of the edge weights which correspond to the number of connections between nodes from module 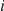 to module 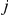, i.e., 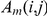.

For each network 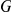 we determine the number of communities 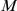 and its modularity value 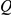 found by application of the partitioning algorithm 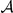 [51]. These measures provide a course overview of the network structure. To obtain more detailed information we calculate 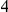 additional measures which are based on the connectivity matrix of the modules, 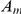, of the module structure of 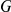. The components of 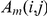, for 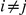 and 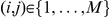, give the number of connections between nodes in module 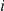 to nodes in module 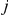. All self-connections, 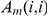, are set to zeros. In the following we consider only undirected networks 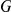, hence, 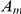 is a symmetric matrix. In addition, we calculate a vector 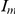 whose components 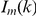 correspond to the number of nodes in module 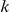. From these auxiliary measures we obtain further measures. We want to remark that the matrix 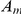 can be considered as (weighted) adjacency matrix of a new network 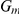, whose nodes correspond to modules. We call 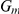 the *module network*. The reason for this is, formally, 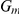 can be seen as the result from a functional mapping from 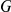, namely, 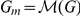. The last equation illustrates that the application of a method for finding a community structure in 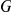 leads to a new network 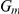. This is illustrated in Fig. 2. The measures defined in the following are calculated for module networks 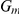 in which one node corresponds to one module, as defined above. Due to the fact that we apply these measures to the module network 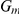, and not to the network 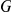, we *enforce* these measures to capture module specific information because 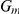 represents explicitly the modules found by the partitioning algorithm 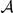.

We define the *relative size*, 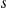, of the largest module with respect to the size of the network,
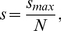
(1)

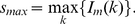
(2)Here 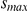 gives the number of nodes found in the largest module and 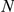 is the total number of nodes in the network 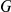. Further, we determine the *normalized entropy of the module connectivity*, 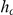, given by
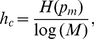
(3)with
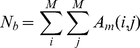
(4)

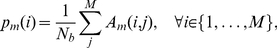
(5)

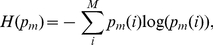
(6)whereby 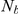 is twice the number of connections between all modules and 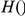 corresponds to the Shannon Entropy [62]. The measure 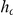 allows to get an impression of the connectivity among the different modules.

Next, we calculate the *mean normalized module-wise entropy*, 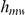, by
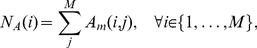
(7)

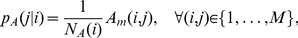
(8)

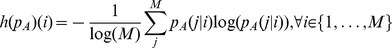
(9)

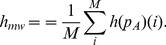
(10)Both entropy measures are normalized because 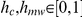, due to the factors that we included in their definitions. The difference between the *normalized entropy of the module connectivity*, 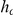, and the *mean normalized module-wise entropy*, 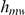, is that for 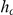 we calculate for each community 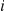 a probability value, 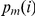, based on its total connectivity to all other modules. In contrast, 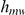 is obtained by calculating a probability vector, 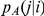, with 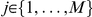, for each community 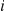. Hence, both measures focus on different structural aspects and for this reason have different discriminative properties with respect to the modular structure of the communities.

Finally, the *normalized mutual information* (nMI) [63] is defined as
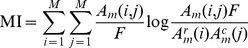
(11)

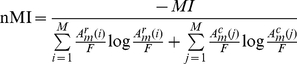
(12)with 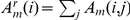, 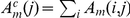 and 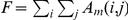. Because the module matrix 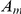 is symmetric for undirected networks 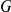, 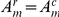 holds. Briefly, Eqn 12 can be written as 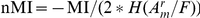, whereas 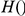 is the Shannon Entropy.

**Figure 3:**
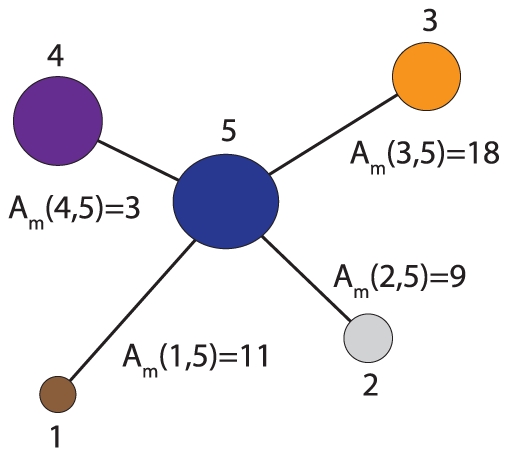
An illustrative examples to demonstrate the usage of our measures (see text). Different modules are shown as colored nodes.

In order to illustrate the numerical usage of our measures we present in Fig. 3 an example. Suppose we have an undirected, unweighted network 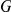 and application of an algorithm 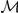 for community finding results in the shown results. Here each node in the network corresponds to a module which may consists of a variable number of nodes, indicated by a varying size of these nodes. Let's call these weighted network 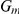, because it describes the structural connectivity among the modules found in 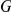. The modules are numbered from 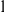 to 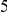 and 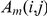 gives the number of connections between module 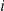 and 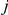. For instance in Fig. 3, module 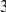 is connected to module 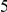 via 18 links. These links are obtained by using the partitioning which is found by application of 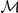 to 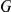, and its corresponding adjacency matrix (not shown). For reasons of simplicity we represent the number of links as weights of edges, instead of 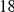 individual links. When two modules are not directly connected then the corresponding component of 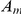 is zero, e.g., 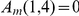. Numerically, we obtain for the example shown in Fig. 3:
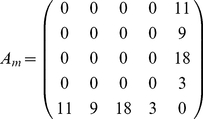
(13)

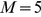
(14)

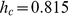
(15)

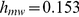
(16)

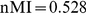
(17)


### Network data and models

Table 1 provides an overview of the 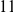 protein interaction networks we are using for our analysis. These data are taken from the BioGrid (BG) and IntAct (IA) database [39], [40]. GCC shown in the fifth column corresponds to the *giant connected component* of the respective network which is the size of a subnetwork with the property that any two nodes are connected via an undirected path. In addition to the protein networks, table 1 contains also three non-biological networks. Specifically, one technological (power grid) and two social networks (netscience and hep-th) are also used in our analysis. ‘Power grid’ is the Western States power grid network, ‘Netscience’ represents a coauthorship network of scientists working on network theory, and ‘hep-th’ is a coauthorship network between scientists posting preprints on the high-energy theory e-print archive. The data for these networks are obtained from [24], [64], [65]. The merit for including these networks in our analysis will become clear in the results section.

**Table 1:** Overview of networks used in our analysis.

network type	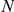	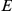	density	GCC
Arabidopsis thaliana (BG)	1675	2953	0.00210	1212
Homo sapiens (BG)	8429	29321	0.00082	8114
Mus musculus (BG)	545	490	0.00330	141
Drosophila melanogaster (BG)	7034	22222	0.00089	6907
Caenorhabditis elegans (BG)	2806	4457	0.00113	2575
Saccharomyces cerevisiae (BG)	5620	53309	0.00337	5611
Schizosaccharomyces pombe (BG)	1411	2478	0.00249	1313
Escherichia coli (DIP)	2856	6712	0.00164	2159
Helicobacter pylori (DIP)	1066	1415	0.00249	976
Mycoplasma pneumoniae (IA)	415	735	0.00855	375
Rattus norvegicus (IA)	1232	1421	0.00187	1095
Western States Power Grid	4941	6594	0.00054	4941
Coauthorship Netscience	1589	2742	0.00217	379
Collaboration Hep-th	8361	15751	0.00045	5835

The first 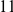 networks are protein networks and the bottom 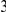 are technological and social networks. The columns refer to the number of nodes (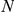) and edges (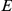) in the network, *density* is the edge density and GCC is the giant connected component. BG: BioGrid database, IA: IntAct database.

We would like to remark that using the GCC of the protein interaction networks has the positive side effect to serve as a denoising of the network data. That means due to the fact that none of the available PPI networks is neither complete (comprises all proteins) nor error free nor unbiased with respect to the coverage of the biological processes, certain parts of the PPI networks are more reliable than others [66]. Here with reliable we mean having a lower error as measured by the false negative and false positive rate of interactions. Due to the fact that the GCC has the property, among others, to be the largest connected subnetwork, it appears to represent such a *lower-error* region in the network compared to, e.g., an unconnected subnetwork composed of many separate protein complexes and interactions. However, an explicit quantification of this effect is currently difficult because it would require to introduce assumptions about the occurring errors and their estimates.

**Figure 4:**
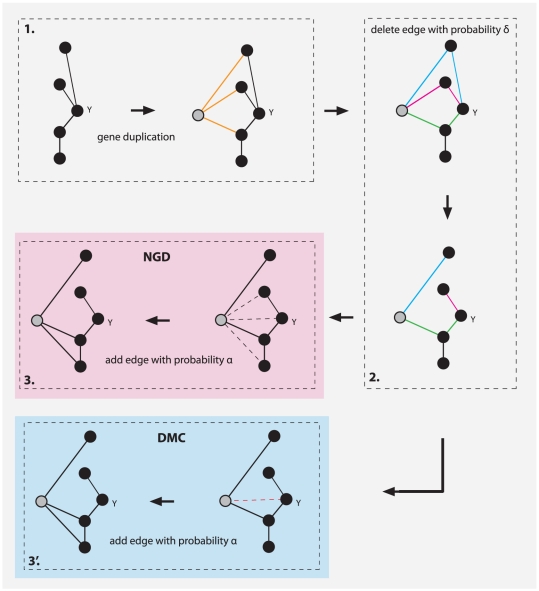
A schematic visualization of the three steps of the *network gene duplication* model (steps 1, 2 and 3: NGD) and the *duplication-mutation complementation* model (steps 1, 2 and 3′: DMC). The colored and dashed edges highlight links or potential links effected with probability 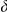 or 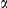.

In addition to the above (real) networks, we generate three types of synthetic networks with three different algorithms. The first two algorithm are very popular models to emulate the evolution of protein networks, namely the *network gene duplication* (NGD) model [36], [37] and the *duplication-mutation complementation* (DMC) [38] model. Briefly, the NGD model starts with a very small number of genes and connections among them, selects one of these genes randomly and makes a copy thereof including its connections to other genes. This corresponds to the introduction of a new gene to the genome. Then two probabilistic mechanisms are applied separately to emulate the divergence of these two genes. The first mechanism consists of a deletion of common links (with probability 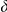) and the second establishes new connections between the new gene and the rest of the genome (with probability 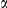). A schematic visualization of the three steps of the *network gene duplication* model are shown in Fig. 4. In the first step, gene 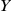 and all its connections are duplicated, resulting in the gene highlighted in grey. In the second step, the three common edge pairs, highlighted in blue, red and green are independently tested and one randomly selected edge of each pair is deleted with probability 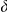. In the third step, the new gene receives with probability 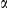 a new interaction to an existing gene. The dashed edges in Fig. 4 correspond to these potential new edges. A summary of the networks and their characteristics we generated with the NGD model, which we use for our analysis, can be found in table 2. In table 3 we provide the corresponding model parameters for their generation. The second algorithm we use models also the evolution of protein networks and is called the *duplication-mutation complementation* (DMC) [38] model. The DMC model is very similar to the NGD model, sharing the first two steps, namely gene duplication and edge deletion; step 1 and 2 in Fig. 4. However, the third step of the DMC model (step 3′) is different, consisting in the connection of the new gene with its template gene with probability 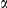. That means this model does not allow to create new connections to other genes already present. Regarding the selection of the parameters of the NGD and the DMC model, we choose 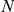 (number of genes) to cover the size of the protein networks we use in our analysis listed in table 1. Also for the probabilities 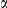 and 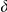 we choose values to result in edge densities that are comparable to the protein networks. In the ‘Results’ section, we provide an additional discussion of the chosen parameters.

**Table 2:** Overview of SCN, NGD and DMC networks.

network type	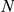	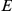	density	GCC
SCN1	2500	8904.6	0.00285	2500.0
SCN2	2500	8995.0	0.00287	2497.4
SCN3	3000	8271.6	0.00183	3000.0
SCN4	3000	12491.2	0.00277	2995.0
NGD1	500	1069.2	0.00857	461.6
NGD2	1000	2183.2	0.00437	826.8
NGD3	1500	7713.4	0.00686	1487.6
NGD4	1500	4495.4	0.00399	1058.2
NGD5	2000	12658.8	0.00633	1988.6
NGD6	2000	14332.2	0.00716	1988.4
NGD7	3000	28013.4	0.00622	2997.2
NGD8	7000	9056.0	0.00369	5826.6
DMC1	3000	5006.0	0.00112	3000.0
DMC2	1000	1423.0	0.00284	1000.0
DMC3	2000	5731.0	0.00286	2000.0
DMC4	3000	5018.5	0.00111	3000.0
DMC5	3000	5003.0	0.00112	3000.0

The shown network measures are averaged over 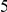 networks.

**Table 3:** Parameters used to generate the NGD and DMC networks shown in table 2.

network name	N	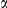	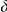
NGD1	500	0.0100	0.75
NGD2	1000	0.0050	0.70
NGD3	1500	0.0070	0.90
NGD4	1500	0.0050	0.70
NGD5	2000	0.0050	0.80
NGD6	2000	0.0050	0.70
NGD7	3000	0.0050	0.80
NGD8	7000	0.0005	0.95
DMC1	3000	0.47	0.80
DMC2	1000	0.22	0.85
DMC3	2000	0.27	0.65
DMC4	3000	0.52	0.80
DMC5	3000	0.12	0.80

The third algorithm we use to generate networks was introduced in [67] for generating a test set of networks with known community structure. The algorithm itself is not based on a biologically plausible mechanism that would correspond to an interpretable genomics mechanism, but serves purely as a benchmark generator. We name networks generated with this method *synthetic community networks* (SCN). In table 2 we show an overview of SCN networks we use for our analysis and table 3 provides the corresponding model parameters.

## Results

In order to perform a numerical analysis to investigate the similarity respectively dissimilarity between protein networks and either synthetically generated or technological and social networks, we calculate for each of these networks 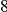 different network-based features, 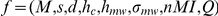, as described in the ‘Methods’ section. All networks we are using in the following analysis are listed in table 1 and 2. For each of the SCN and NGD networks we generated 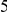, and for each of the DMC parameter settings 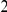 different networks in order to capture the variability of the stochastic process underlying each of the three network models. This gives a total of 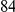 networks we are using in our study, namely, 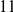 PPI networks, 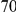 synthetic and 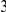 technological and social networks.

**Figure 5:**
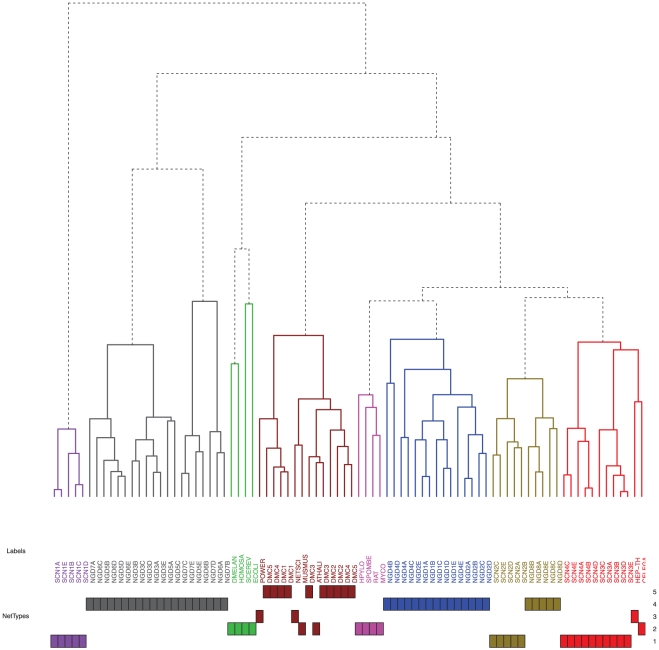
Hierarchical clustering of all 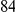 networks used in our analysis. The color of the eight clusters from left to right (for a discussion see text): purple, gray, green, brown, magenta, blue, gold, red. The ‘NetType’ refers to the four principle network types: 1- synthetic networks (SCN), 2 - PPI networks, 3 - non-biological networks, 4 - network gene duplication networks (NGD), 5 - duplication mutation complementation networks (DMC).

From the 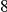 features 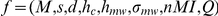, we identify by an exhaustive search the best performing subset with the highest discriminatory power to separate PPI networks and gene duplication networks from each other. From this analysis, we found the 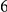-dimensional feature vector
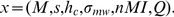
(18)to perform best. The resulting hierarchical clustering is shown in Fig. 5. As distance measure for the feature vectors we used the Canberra distance [68]

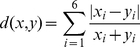
(19)and for the agglomerative clustering we used the Mcquitty method [69] which joins clusters if they are reciprocally similar to each other.

The dendrogram in Fig. 5 consists of 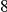 principle clusters (branches), each consisting of related networks, e.g., SCN, NGD, DMC or PPI networks. The colors of these eight clusters are from left to right: purple, gray, green, brown, magenta, blue, gold and red. For example, the left most cluster (purple) contains only SCN networks of various types (as defined in table 3). Similarly, the gray and the blue clusters consist only of NGD networks (for parameters see table 4). Also the PPI networks form two distinguished clusters shown in green and magenta, containing 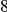 of the 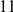 PPI networks. Interestingly, the remaining 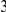 PPI networks are clustered together with the 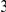 non-biological networks, namely, the coauthorship networks (Netscience and Hep-th) and the power grid (brown and red cluster). We want to remark that we repeated the above analysis also for other clustering methods, e.g., complete-linkage and Ward's method, and received qualitatively similar results to the presented ones.

**Table 4:** Parameters used to generate the synthetic community networks (SCN) shown in table 2.

network name	N	k	maxk	mu	minc	maxc
SCN1	2500	6	150	0.10	4	50
SCN2	2500	6	180	0.15	4	200
SCN3	3000	6	50	0.10	20	50
SCN4	3000	8	60	0.05	10	150

For explanation of the parameters the reader is refered to [67].

From the dendrogram in Fig. 5 follow two important observations. First, the 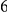 network-based measures we employ to characterize the module structure of a network, result in feature vectors that allow a very good separation of the different network classes (populations). The class of a network can be seen as a label that assigns a network to a specific network category. Due to the fact that clustering analysis represents a form of unsupervised learning [70] these network labels haven't been used for this analysis. Hence, our feature vectors would allow to discover network classes, without a training sample, in a predictive manner. This demonstrates that our feature vectors, respectively the network-based measures, capture sensible information about the module structure of the networks that corresponds with an intuitive separation of them. Here, it is important to emphasize that the resulting clustering provides the intuitive grouping of network classes, although, the components of the feature vectors are abstract entities which may have no intuitive appeal at first sight. It is also interesting to highlight that the protein interaction network of *Mus musculus* is closest to the coauthorship network ‘Netscience’. From table 1, one can see that the GCC of *Mus musculus* is the smallest of all PPI networks and, hence, a largely incomplete network, because the available PPI network contains 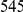 proteins of which only 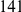 are in the GCC (see table 1), whereas the estimated number of genes for mouse is 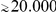. Further, *Arabidopsis thaliana* is the only PPI network from a plant in our analysis. This distinctiveness may explain the separation of these two PPI networks from the two principle PPI clusters in Fig. 5 (green and magenta cluster). On the other hand, due to the fact that the PPI networks of *Mus musculus* and *Arabidopsis thaliana* are not randomly scattered in the dendrogram, there seems to be a clear signal with respect to the underlying characteristics of the population of PPI networks that is detactable by our feature vectors, even for such atypical networks. This is also the reason for using the PPI networks of *Mus musculus* and *Arabidopsis thaliana* in our analysis, because it allows for an indirect test of our assumption we made about the GCC, as explained in the section ‘Network data and models’.

The second interesting observation from Fig. 5 is that the networks generated with the *duplication-mutation complementation* (DMC) model are similar to 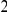 of the 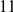 PPI networks, namely the PPI network of *Mus musculus* and *Arabidopsis thaliana* (brown cluster). In contrast, the networks generated with the NGD model, appear not to resemble important structural characteristics of the PPI networks, because otherwise these networks would not be assigned to distinct branches of the clustering but would be found in the same clusters as the PPI networks. Here, the fact that there *exist* parameter settings of the *network gene duplication* model that lead to very different network structures which can be discriminated easily, as the purple cluster on the left-hand side shows, is not as important as the *none existence* of a parameter setting that would lead to a common cluster consisting of PPI and NGD networks.

We tried many different combinations of probabilities to add and delete links for the NGD and DMC model, as controlled by the two model parameters 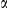 and 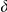, to explore the parameter space of these two models, however, none resulted in clusters that would be significantly different from the presented ones. Another interesting observation in this respect is that the second type of synthetic networks, indicated by SCN2, leads to network structures that are quite similar to the NGD8 networks (gold cluster). This is interesting because the model that underlies the SCN networks hasn't been conceived with the purpose to produce biologically plausible results. Instead, the underlying idea was solely to generate a set of benchmark networks with a know module structure [67].

It is amazing to see that the coauthorship network (Netscience) and the power grid resemble the module structure of PPI networks similarly good as the networks generated with the DMC model. This motivates also the reason for including them in the analysis because this finding hints that *naturally generated* networks are significantly different to *mathematically constructed* networks. Here, we consider the SCN, NGD and DMC networks as mathematically constructed.

### The contribution of individual structural features

The above analysis is based on a 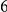-dimensional feature vector, namely, 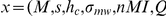). In order to gain insights into the differences of the structural properties of PPI networks and gene duplication networks, we conduct an analysis to estimate the contribution of individual features to the separation of these networks. We start from a set of 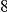 different features (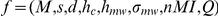) and eliminate subsets thereof. Specifically, we eliminate up to 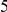 features from (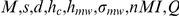) which gives a total number of
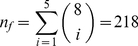
(20)different feature vector combinations, 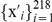. Here, a 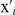 corresponds to such a feature vector. The hierarchical clustering for each of these 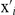 is assessed by a *clustering score*. This score is additively defined over all branches in a hierarchical clustering, whereas for each branch we calculate the product of the number of PPI networks and the number of NGD and DMC networks. That means branches with a mixed population of networks have a high score and branches with only one network type have a score of zero. Coarsely speaking, the clustering score quantifies the mixing of the PPI and gene duplication networks and a perfect score of zero corresponds to a perfect separation of these networks.

**Figure 6:**
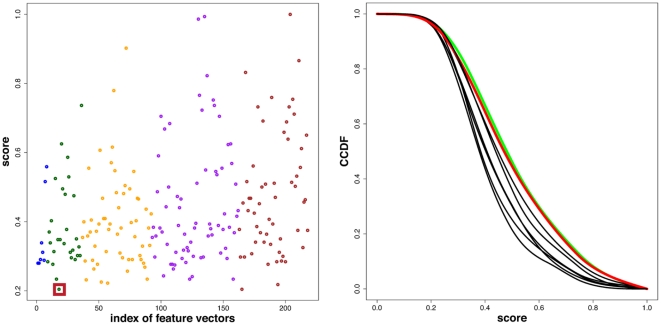
Left: Clustering score in dependence on the index of the used feature vectors. The colors indicate the number of features used for the hierarchical clustering. From left to right: blue (7 features), green (6), orange (5), purple (4), brown (3). The red surrounded dot (index 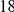) corresponds to the lowest score that was obtained for the features: 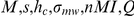. Right: Complementary cumulative distribution function (CCDF) for each of the eight features in dependence on the score.

The results of this analysis is shown in the left Fig. 6. Here the x-axis corresponds to the indexed feature vector combinations, 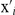, and the y-axis is the normalized clustering score. The color of the dots indicate the number of features used for the hierarchical clustering. From left to right: blue (7 features), green (6 features), orange (5 features), purple (4 features) and brown (3 features). From Fig. 6 one can see that there is just one feature vector combination that leads to a minimal score of 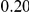, which means that there only a few branches in the hierarchical clustering where PPI and gene duplication networks can be found together. This corresponds to the hierarchical clustering shown in Fig. 5.

To obtain a quantification for the contribution of individual features on these results, we conduct the following analysis. First, we estimate for each of the 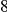 features 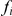 its score density, i.e., 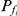. That means if a feature has *not* been used for a clustering, we use the obtained score for this clustering for a density estimation. Then, we calculate from the score densities the *cumulative distribution function* (CDF), 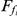, and the *complementary cumulative distribution function* (CCDF),
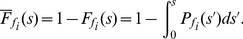
(21)The meaning of 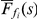 is as follows: If feature vector 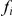 is *not* part of a feature vector combination than the probability of observing a score larger than 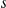 is 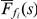, i.e.,
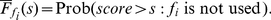
(22)The results for the 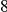 different complementary cumulative distribution functions are shown in the right Fig. 6. For any selected value of the score, one observes always the same two CCDFs with the highest probability, highlighted in green and red. These two CCDFs belong to the features 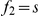 (green) and 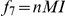 (red). Hence, the *absence* of these features in a feature vector leads always to a higher probability to observe higher clustering scores. In other words, if the features 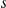 or 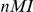 are not considered for a hierarchical clustering, the discriminative power of any feature set is compromised. However, due to the closeness of all 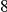 CCDFs, see Fig. 6, this effect is not strong enough to claim that only these two features are sufficient to result in a clustering with a low or even the lowest score. This is confirmed by a numerical analysis which gives a score of 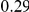 for the feature vector 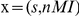.

This analysis demonstrates that there is no individual structural property in a network that has enough discriminatory power to allow separating branches of PPI and gene duplication networks. Instead, the combination of a variety of different features is needed.

### Connecting data with models

**Figure 7:**
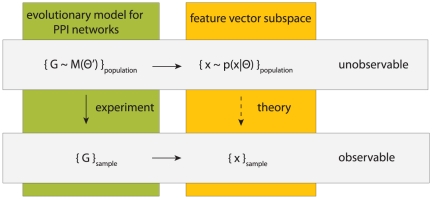
A schematic visualization of the connection between the underlying model 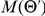 to generate networks and the feature vectors 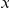, used in our study.

Finally, we discuss the rational behind our analysis, which will also shed light on the robustness and interpretation of our results. In order to simplify the following discussion, a visual summary is presented in Fig. 7. First, we assume the existence of a (evolutionary) process that leads to the emergence of different species. For our discussion a species is represented by its underlying PPI network. An evolutionary process we consider abstractly as a model 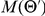, which, depending on a set of parameters 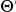, generates PPI networks. The entity of all possible PPI networks that can be generated from the model 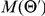 constitutes the population of PPI networks. From each PPI network 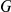 we can derive a feature vector 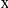, of a certain dimension, whose components represent properties of 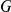. This leads to the population of feature vectors that represents these properties for the whole population of PPI networks. It is important to note that we assume the dimension of 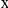 to be finite. For this reason, there is an unidirectional mapping from 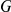 to a feature vector 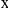 which means that the properties given by 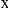 may not be sufficient to reconstruct the network itself. Theoretically, one can assume that these feature vectors are drawn from an unknown probability distribution, 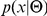. However, neither 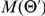 nor 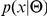 are known or observable. The data available for an analysis are the PPI networks from a (small) number of different species. These PPI networks can be seen as a sample from the population of all possible PPI networks from all species obtained experimentally. From this sample of networks, one can derive feature vectors which constitutes theselves a sample. We would like to note that the sample of feature vectors 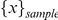 is only given indirectly via the observation of 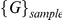 because 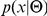 is not accessible experimentally. In summary, we assume a mapping from an evolutionary model that constitutes the population of PPI networks, which is unobservable, to a sample of feature vectors, each representing one species. The practical merit from this mapping is that feature vector can be statistically studied within the framework of multivariate analysis. The model visualized in Fig. 7 describes exactly the way feature vectors were obtained for both synthetically generated network models. For example, in the case of the *network gene duplication* model we have 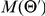 with 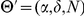.

From the above model follow several implications that are important for the interpretation of our results shown in Fig. 5. First, we do not need to make detailed assumptions about the evolutionary model. That means it could be just one model or three different models, e.g., one for Bacteria, Archaea, and Eukaryota. Instead, it is enough to assume that a PPI network is represented by a random vector. From the clustering of feature vectors shown in Fig. 5 one can see that the 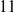 different PPI networks are not randomly scattered, but clustered together in 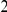 clusters. This is an indicator that despite the differences that certainly exist among the individual PPI networks, respectively the underlying species, they are more similar among each other than with other network types. As a side note, this could imply that they are describable by just one underlying evolutionary model 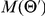 to represent all PPI networks, but with different parameter values for different species. The fact that the PPI networks are not clustered in just one branch is no counter argument against this, because also the SCN and NGD networks, of which we know they are generated from the some underlying model, are distributed over several clusters.

More important for our analysis is the effect of the errors in the PPI networks. It is clear that none of the available PPI networks is error free, either missing true positive connections among proteins or, probably less likely, included false positive connections. In order to estimate these errors explicitly one would need to introduce a specific error model, which is based on assumptions. However, our framework does not require us to explicate such assumptions. More specifically, it is known that the errors in the PPI networks (false positives, false negatives) were created in a biased manner [66] effecting all networks. With respect to Fig. 5 this corresponds to a mechanism that influences the mapping
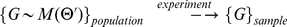
(23)in the following way
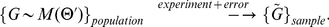
(24)This leads to the actually observed networks 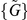, which are different to the (true) PPI networks 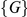. Due to the fact that our analysis is *comparative*, based on a hierarchical clustering, investigating networks with respect to their similarity to other networks rather than individually, the effect of the presence of errors in the PPI networks is alleviated, when all PPI networks are approximately homogeneously effect by errors. If the differences between the errors on the feature vectors would be severely heterogeneous, we would not be able to observe clustered PPI networks, but they would be randomly scattered in the dendrogram. Hence, the presence of clusters of PPI networks in the dendrogram supports the claim that all PPI networks are similarly effected by errors and also that the noise level in these networks is smaller than the signal, as captured by the feature vectors, because otherwise there would be no meaningful clustering possible leading to discernible separations of networks of different classes.

## Discussion

In this paper, we studied the question if *gene duplication models* allow to generate networks with a module structure that resembles the module structure one can find in experimentally obtained protein interaction networks. The results from our clustering analysis revealed the existence of different structural features on the module level, the NGD model and the DMC model exhibit compared with biological protein interaction networks and, hence, demonstrate limitations of these models [71]. We want to emphasize that we studied not only the parameter settings of the models listed in table 2, but many more. However, none resulted in qualitatively different results. This points to a general limitation of these models in the description of the evolution of modules in protein networks.

We would like to highlight that we found from our analysis the *duplication-mutation complementation* model [38] to be a better model for PPI networks than the *network gene duplication* model, at least for the two protein networks of *Mus musculus* and *Arabidopsis thaliana*. This difference in these two models hints that the new establishment of functional interactions between a new gene and genes already present in a genome (step 3 in Fig. 4) are less beneficial than their neglect (setp 3′ in Fig. 4). Hence, this information could be utilized to revise an evolutionary duplication model, e.g., in combination with other biological mechanisms. For example, higher-order extensions with respect to the number of duplicated genes may be needed to rectify the obtained module structure of the resulting networks. Biologically, there is a multitude of genomic mechanism ranging from whole-genome duplication (polyploidy) to a restricted duplication of chromosomal regions [43], [73]–[75] that provide ample opportunity for exploring plausible modifications of an extended duplication model.

From analyzing the influence individual features have on the separation of PPI and gene duplication networks, we found that there is no single structural feature that possess sufficiently discriminatory power to accomplish such a separation, but one needs a combination of several features. As most influential features we identified the relative size of the largest module (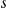) and the normalized mutual information (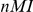). Interestingly, for PPI networks one finds a negative correlation coefficient of 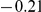 between the values of 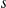 and 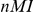. For NGD networks their correlation is 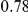 and for DMC networks 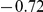. Further, the correlation between the number of modules (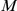) and the modularity (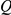) is for PPI networks negative (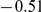) and for NGD and DMC networks positive (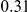 and 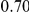). This indicates structural differences of modules in the two gene duplication models, but also between these models and the PPI networks. Interestingly, for gene duplication networks an increasing number of modules (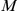) is associated with an *increasing* modularity (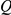), whereas for PPI networks it is associated with a *decreasing* modularity.

The absence of individual features, allowing a separation of PPI and gene duplication networks, is not surprising because the module structure of networks corresponds to a mesoscopic level of description. This implies that *systems properties* are playing an important role which can not be reduced to individual proteins or features. Hence, our results are in accordance with the view of evolutionary systems biology [72] considering evolution as a high-dimensional process.

The NGD model and the DMC model have been studied numerously over the last few years and demonstrated to reproduce several features that are in accordance with protein networks [35], [76]–[80]. However, these studies focused either on global properties of protein networks, e.g., degree distributions, studied individual protein networks only or investigated network motifs. Instead, in this paper we studied the module structure of protein interaction networks, which is generally believed to play a key role in the functional understanding of an organism. Another difference is that in our analysis we did not focus on individual protein networks but we considered all protein networks to belong to a population (or a sample thereof). This is an important difference because it allows to capture biological variability that is inevitably present in protein interaction networks from different organisms as well as in any stochastic process that generates networks like the NGD or DMC model. Hence, conducting a *comparative*, instead of an *individual* protein interaction network analysis allows to borrow strength from different members of the population (sample) to alleviate errors. As a direct consequence thereof, the basic entities of our analysis are the resulting branches of the clustering and their composition and not the position of individual networks. A related, yet different aspect of our approach and the fact that a clustering analysis performs a comparative analysis is that our study does not aim to provide precise estimates for specific network statistics, e.g., by means of interval estimators and their corresponding confidence intervals. The latter would require the introduction of additional assumptions and a different methodology, specifically adopted to the characteristics of the studied networks; see [81] as an example for such a study. In general, a clustering analysis is considered as an exploratory analysis which provides a valuable comprehension into the pattern of data without the need of making strong assumptions [82]. Hence, the simplicity of our approach is that it requires only a minimum of assumptions compared to more elaborate methodological approaches, e.g., confirmatory methods [83], and, hence, constitutes in the light of our limited knowledge about the evolution of protein interaction networks a sensible first step to gain insight into the complex and important module structure of protein networks.

We would like to emphasize that the modularity of a network is just one property of a network, like the degree distribution or the average path length. For this reason, it would be interesting to study further network properties of the *network gene duplication* model, the *duplication-mutation complementation* model and protein interaction networks to see if there are additions differences between these networks. Using the module structure was guided by biological considerations, however, one could also approach this problem from a more theoretical perspective probing different network characteristics. If such an abstract distinctive network property could be found, it would be interesting to think about a biological elucidation for this effect. The potential gain from such an analysis could be to discover novel biological features that may have been overlooked so far, because only properties with a clear biological interpretation have been studied.

Despite the fact that the primary concern of this paper is a biological topic in evolutionary biology the similarity of module structures between PPI networks and the coauthorship networks (Netscience and Hep-th) and the power grid is interesting. It hints that much can be learned from analyzing networks from different origin and different disciplines [84].
