# Correction: Limitations of Gene Duplication Models: Evolution of Modules in Protein Interaction Networks

**DOI:** 10.1371/annotation/4f3cfa51-aac6-4ffe-80f1-5ec467921edd

**Published:** 2012-06-07

**Authors:** Frank Emmert-Streib

Figure 2 in the PDF does not precisely match the image of Figure 2 in the online version of the article. The online version is correct. 

